# Performance analysis of 349-element adaptive optics unit for a coherent free space optical communication system

**DOI:** 10.1038/s41598-019-48338-3

**Published:** 2019-09-11

**Authors:** Leqiang Yang, Kainan Yao, Jianli Wang, Jingtai Cao, Xudong Lin, Xinyue Liu, Wei Liu, Haijun Gu

**Affiliations:** 1 Changchun Institute of Optics, Fine Mechanics and Physics, Chinese Academy of Sciences, Changchun, Jilin 130033 China; 2 University of Chinese Academy of Sciences, Beijing, 100049 China; 3 College of communication Engineering, Jilin University, 5372 Nanhu Road, Changchun, 130012 China

**Keywords:** Atmospheric optics, Fibre optics and optical communications

## Abstract

As a continuation of our previous work [Optics Express.25, 15229(2017)] in which we have verified the performance of a coherent free space optical communication (FSOC) system with a 97-element adaptive optics (AO) system, in this paper, we evaluated the performance improvement of the coherent FSOC system using a large-scale high-speed AO system with a 349-element continuous surface deformable mirror. The mixing efficiency (ME) and bit-error-rate (BER) under different Greenwood frequency (GF) were calculated as the performance metric of coherent FSOC system. The performance of FSOC system using such a large-scale AO system was quantitatively verified for the first time. The obtained results showed that the performance was obviously improved when a larger-scale high-speed AO system is employed in coherent FSOC system. This analysis result provides a performance verification for large-scale high-speed AO systems used in FSOC system which is beneficial for coherent FSOC system parameters design.

## Introduction

Free space optical communication has been considered as an effective solution for both ground to satellite transmission link and last-mile applications due to its high bandwidth, excellent security, free license spectrum and low cost^1,2^. Especially in ground-to-satellite communication links, FSOC technology has been successfully deployed in many applications^3–5^. Comparing with the traditional intensity modulation direct detection scheme, the coherent detection has attracted more and more attentions due to its high sensitivity and high communication data rate^5–8^. However, a major problem for the coherent FSOC system is the beam propagation through the atmospheric turbulence which often brings intensity scintillation and wavefront phase distortion at receiver thus degrades the performance of coherent FSOC system^1,9–11^. Many institutes and researchers have proposed many effective solutions to overcome the performance degradation caused by turbulence^6,12^. For ground-to-satellite applications, the scintillation effects are usually weak while the wavefront distortions are the major concern that cause the performance degradation^4,12^.

Adaptive optics is considered as one of the effective methods to improve beam quality by rapidly compensation the atmosphere-induced wavefront aberrations and has made great achievements in many applications^5,6,13–16^. To realize better performance in optical communications, the adaptive optics system must be designed to correct both high spatial and high temporal frequency wavefront aberrations to allow near-diffraction-limited operation at the receiver^17^. In a typical AO system, the inherent ability of AO system to correct high spatial and temporal frequency aberrations are determined by the element number of deformable mirror (DM) and the closed-loop control bandwidth (CLCB), which represent the spatial characteristic and the temporal characteristic of AO system respectively. Different choices of these parameters will lead to different performance of the coherent FSOC system. It is a very important issue in the coherent FSOC system design and has been under rapid progress in recent years. Ming Li *et al*. studied the performance of coherent FSOC system in the case when the quadrature phase-shift keying modulation format is applied and when a 108-element AO system is employed^18^. The obtained results illustrated that the performance of coherent FSOC system is greatly improved with the use of AO system. Yukun Wang *et al*. investigated the influence of the spatial and temporal characteristics of turbulence on the performance of AO in a FSOC system using a 145-element deformable mirror^19^. Chao Liu *et al*. investigated the mixing efficiency and BER performance improvement of the coherent FSOC system with the use of a 127-element AO system mounted on a 1.8 m telescope^5,8,20^. The experimental results showed that the AO technique is a powerful tool and has great potential to improve the performance of coherent FSOC system. In 2018, their group verified the relationship between the zenith angle and performance of coherent receiver^4^. The experiment was carried out with a 249-element AO system incorporated with a 1.8 m telescope as same as before. The results demonstrated that AO system has a significant improvement on coherent FSOC system at a large zenith angle. However, the results also indicated that the 249-element AO system cannot guarantee the FSOC performance when the zenith angle was larger than 65°. As for the temporal characteristics, the relationship between the servo bandwidth of AO system and Greenwood frequency for coherent FSOC system was investigated using different servo bandwidth values^7^. Now it is well known that more element number and higher CLCB will get a better performance. However, limited by cost and the system complexity, the quantitative relationship between the performance of coherent FSOC system and larger scale higher speed AO system has not been validated by experiments. Therefore, the performance analysis of FSOC system using large-scale AO system is required to guide the parameter design of AO system used for FSOC system.

In this paper, as a continuation of our previous work^13,21^, a higher speed AO system with a double-stage fast-steering-mirror (FSM) and a 349-element continuous surface deformable mirror (CSDM) was introduced in coherent FSOC system to compensate higher spatial and temporal frequency wavefront aberrations. To the best of our knowledge, this is the AO system with the largest element number for coherent FSOC system performance analysis. The mixing efficiency and bit-error-rate were analyzed to evaluate the performance of the coherent FSOC system. The numerical simulation was used to illustrate the performance improvement of coherent FSOC system can be increased by increasing the element number of DM. Furthermore, an experimental system was designed to evaluate the improvement of the coherent FSOC system performance with a 349-element high-speed AO system. The conclusion of this paper can provide a guideline for the design of coherent FSOC system with similar configuration of AO system.

## Results

### Simulation results

For this numerical study, we simulate the theoretical performance for a given coherent FSOC system under different turbulence conditions. The communication wavelength of the coherent FSOC system is 1550 nm, the receiving telescope diameter (D) is 1.2 m. We assume the atmospheric coherent length (*r*_0_) is 7 cm, thus the corresponding ratio of receiver aperture diameter to atmospheric coherent length (*D*/*r*_0_) is fixed at about 17, which is a relatively strong turbulence condition. Since the turbulence influence are similar for homodyne and heterodyne detection, the homodyne detection scheme is selected to analyze, and the intensity scintillation is also ignored which means that the amplitude distributions of the received optical signal and the LO laser are uniform. In the previous work, we had confirmed the relationship between the performance of coherent FSOC system and the CLCB of AO system under different Greenwood frequency and *r*_0_^13^. In this paper, we will focus on the relationship between the element number of deformable mirror and the performance improvement of FSOC system.

**Figure 1:**
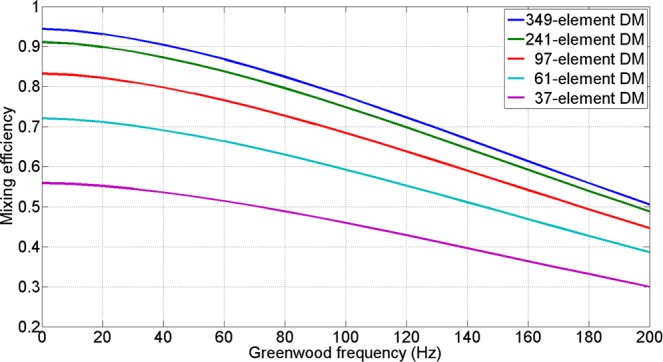
The mixing efficiency versus Greenwood frequency for different element DM.

According to Eq. 10 in Method section, Fig. 1 is a plot of the mixing efficiency as a function of Greenwood frequency for different configurations of AO system. The element number for each configuration is from 37 to 349 and the corresponding equivalent distance between the actuators projected on the entrance pupil of the 1.2 m receiving antenna is from 24 cm to 6 cm respectively. And the CLCB of AO system is fixed at 120 Hz. As shown in Fig. 1, the mixing efficiency decreases rapidly with the increase of Greenwood frequency. And when Greenwood frequency is zero, which means spatial error is the only error source, the mixing efficiency of a 61-element deformable mirror AO system is 0.72 and the value of 37-element deformable mirror is only 0.55 which is even smaller. This simulative result is due to our simulation condition is a relatively large diameter telescope and a strong turbulence condition, thus the spatial frequency of wavefront aberration is relatively high. Only using a 37-element DM or 61-element DM cannot compensate the high spatial frequency aberration very well.

With a 97-element AO system correction, the mixing efficiency increase to 0.83 or 0.68 when Greenwood frequency equals to 0 or 100 Hz, respectively. When the Greenwood frequency is low (lower than 40 Hz), the mixing efficiency can reach higher than 0.8. And with the increase of Greenwood frequency, the mixing efficiency degrades significantly. When Greenwood frequency is more than 140 Hz, the mixing efficiency is below 0.6. And with a 349-element deformable mirror which can compensate high spatial frequency wavefront aberration better, when Greenwood frequency is 0 or 100 Hz, the mixing efficiency can reach 0.94 or 0.78, respectively. It is obvious that increasing element number of DM can improve the ability of AO system to suppress the high spatial frequency aberrations. When Greenwood frequency is higher than 200 Hz, the mixing efficiency cannot be over 0.5 since the temporal error become the dominant factor which degrades the mixing efficiency.

**Figure 2:**
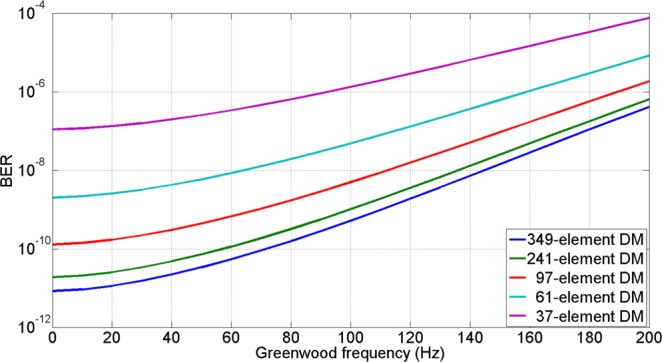
The BER versus Greenwood frequency for different element DM.

According to Eq. 14, when the number of photons per bit equals to 12 and the quantum efficiency of the detector is deemed to 1, the relationship between BER performance and Greenwood frequency is shown in Fig. 2. As shown in Fig. 2, when a 37-element or 61-element deformable mirror is employed, it is unable to meet the BER of less than 10^−9^. With a 97-element AO system, when the Greenwood frequency is below 70 Hz, the BER value can be suppressed to less than 10^−9^. However, when the Greenwood frequency is more than 80 Hz, the 97-element AO system cannot guarantee a BER below 10^−9^ either. As for a 349-element AO system, it can meet the requirement when the Greenwood frequency is below 110 Hz, which means it can adapt to more severe atmospheric conditions and has better performance than the other systems.

Depending on the above analysis of the simulative results, it is obvious that the element number of DM has significantly influence on the performance of coherent FSOC system with AO correction. When the element number of DM increases from 97 to 349, the BER values can decrease about one order of magnitude on average.

### Experiment results

**Figure 3:**
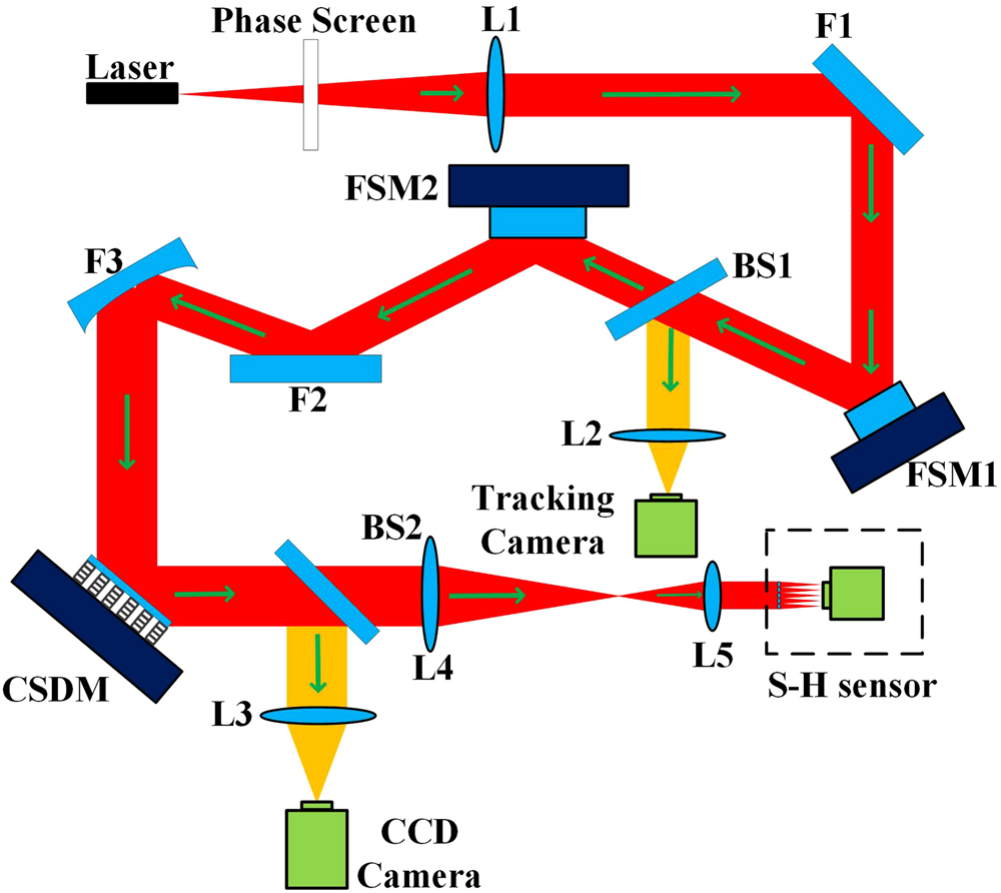
Structure of our experiment system.

In order to explore and verify the performance improvement of coherent FSOC system under different Greenwood frequency, a lab experimental AO system with a double-stage FSM and a 349-channels CSDM was conducted as shown in Fig. 3. The aperture of the matching telescope is 1.2 m.

**Figure 4:**
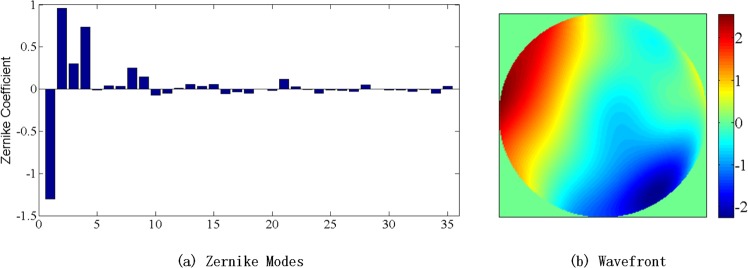
Zernike coefficients and wavefront of the phase screen.

As shown in Fig. 3, the light beams first pass through a phase screen which was built by Lexitek company and satisfies the Kolmogrov turbulence theory. By changing the rotating speed of the phase screen, we can simulate turbulence with different Greenwood frequency. A typical wavefront distorted by the phase screen can be seen in Fig. 4. Figure 4(a) describes the Zernike coefficients of the phase screen at a certain time and Fig. 4(b) is the corresponding wavefront. The RMS value of the wavefront at this certain time is 1.03*λ*(*λ* = 1550 *nm*). And it can be seen from the Zernike coefficients and the wavefront that the aberration induced by atmosphere can be well simulated by the phase screen.

Then the beams are expanded and collimated by lens L1 and the off-axis parabolic reflective mirror F1, and then entry the AO system. The aperture of the collimated beams is 56 mm, slightly smaller than the effective aperture of the FSM. The first part of the AO system are the double-stage FSMs, which provide tip/tilt correction. Both two FSMs with large stroke and high speed are same and made by PI Company. The FSM1 has a physical range of +/−1 mrad and is driven by a high speed tracking camera operates at 400 Hz to measure the tip-tilt aberration. The tracking camera is an Andor iXon860 with a pixel size of 24 *μm* and the camera resolution is 128 × 128. Besides, the focal length and the effective aperture of focusing lens L2 are 240 mm and 56 mm respectively. The FSM2 is driven by the Shack-Hartmann(S-H) wavefront sensor which will be detailedly described in the following paragraph, and it can measure high frequency tip/tilt aberration due to the high frame frequency of S-H wavefront sensor.

**Figure 5:**
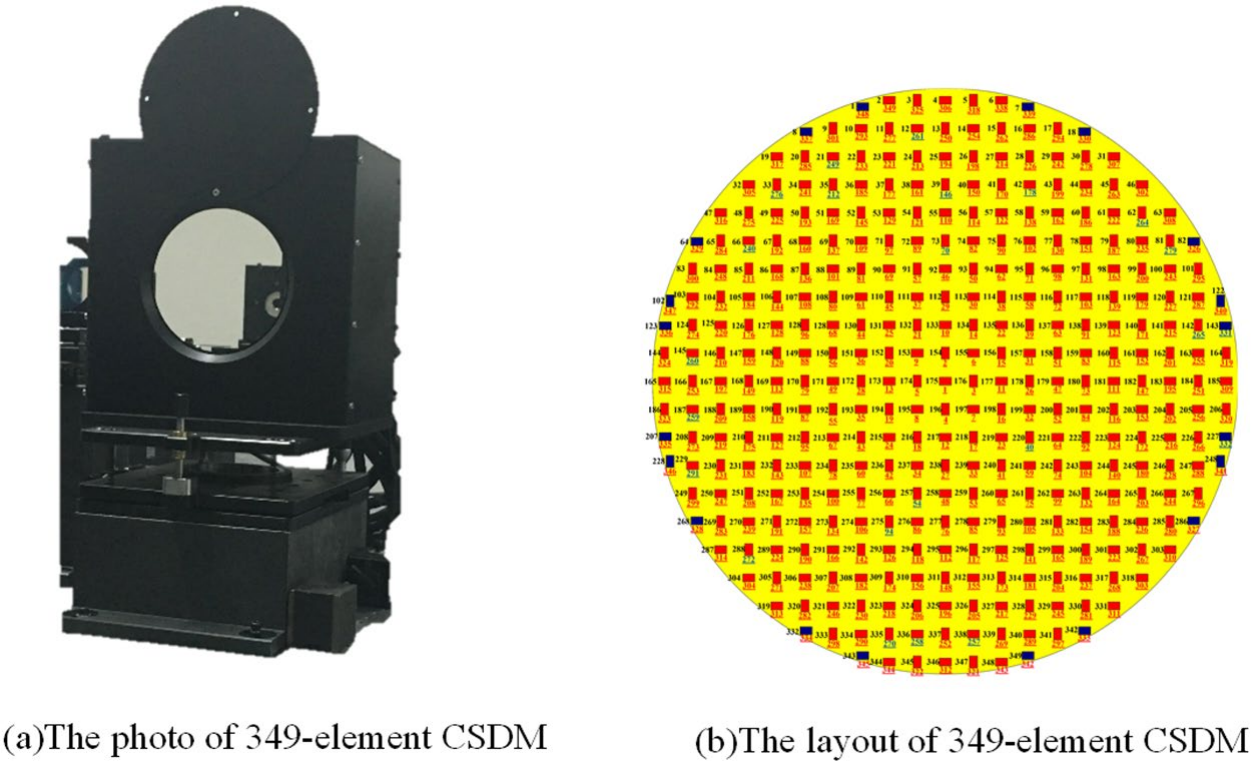
The photo and layout of 349-element CSDM.

The second part of the AO system is composed of the 349-element CSDM, the Shack-Hartmann wavefront sensor and the control system, which are used to measure and compensate the dynamic high-order aberrations. The CSDM is a $\varphi \,150\,mm$ continuous surface deformable mirror which was custom designed and manufactured by ourselves. It consists of 349 piezoelectric ceramic actuators with a reflective surface bonded to the top of the actuators structure with a silver coating. The size of actuator is 2 mm and the grid spacing is 7 mm, and its configuration is described in Fig. 5. The full diameter of the mirror contains 21 actuators and each actuator has a full stroke of 5 *μm* and is driven by a voltage ranged from 0 to 120 V. The DM electronics have a bandwidth of 5 kHz so that no significant delay is introduced by the electronics. The surface RMS is about 700 nm before self-flattening and about 14 nm after self-flattening.

The S-H wavefront sensor is composed of micro lenslet array and high speed electron-multiplying charge-coupled device (EMCCD) camera. A beam compressor composed of L4 and L5 is deployed to image the CSDM onto the lenslet array and reduce the beam size to match the CSDM actuator spacing to the pitch of the lenslet array. The focal length of L4 and L5 is 608 mm and 52 mm, respectively and the effective aperture size of L4 and L5 is 56 mm and 12 mm respectively. The EMCCD is an 240 × 240 pixel OCAM2 camera with a 24 *μm* pixel size manufactured by FirstLight Company^22,23^. It can operate at 1500 Hz with a readout noise lower than 1*e*^−^. The S-H wavefront sensor has 349 effective sub-apertures in a 21 × 21 square. And the aperture size and the focal length of micro-lenslet array are 200 *μm* and 7 mm respectively.

**Figure 6:**
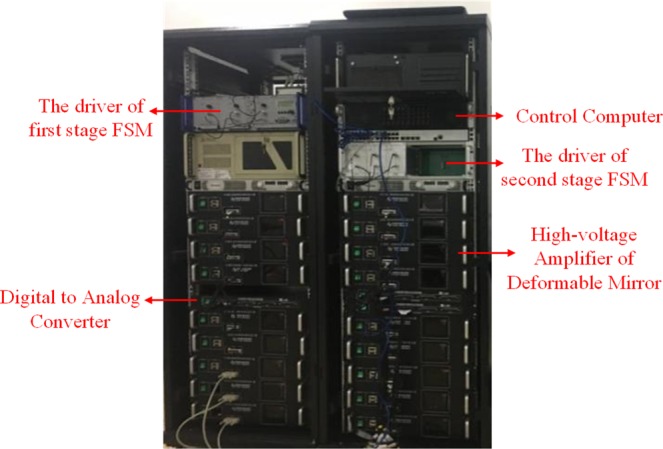
The control system of AO unit.

As shown in Fig. 6, a control system is employed to receive the wavefront aberration information measured by Shack-Hartmann wavefront sensor and control the high-voltage amplifier to drive the deformable mirror generating a phase surface conjugated to the measured one accordingly. The classical PI control algorithm is adopted and in order to improve the CLCB of AO system, an architecture consists of FPGA and GPU is also employed to reduce the processing delay. Both FPGA and GPU have the powerful parallel processing ability and can remarkably reduce the computation delay^24–26^. Typically, the AO wavefront correcting is performed in two steps: calculation of the wavefront slopes by calculating the gravity of the spots array and calculation of the steering commands for deformable mirror. In our architecture, FPGA is used to calculate the centroids of the spots array from the Shack-Hartmann image which transmitted through a full-mode camera-link lines. It can concurrently calculate the centroids while the Shack-Hartmann camera reads out, thus form a processing pipeline and reduce the processing delay. As an intermediate result, centroids data is transmitted to GPU through Gigabit ethernet using UDP communication protocol. Since the centroids data size is relatively small, only a few KB, so the communication delay is also very small and can be treated as negligible. GPU is in charge of the calculation of the steering commands by multiplying the centroids data with the control matrix. For large scale AO systems, this computation takes the majority of the processing time while it is very suitable to be implemented on GPU since GPU has powerful parallel computation resources and matrix-vector multiplication is a typical problem that can be processed parallelly. After that, the steering commands are sent to the digital to analog converter card via Gigabit ethernet UDP packages as same as the centroids data. Also, this communication delay can be treated as negligible since the data size is very small. According to our measurement, the processing delay was about 220 *μs* which is a relatively small value for the S-H wavefront sensor operating at 1500 Hz. In addition, digital to analog converter is used to convert the digital output control command to the analog voltage signal which is received by high-voltage amplifier to reshape the deformable mirror accordingly.

**Figure 7:**
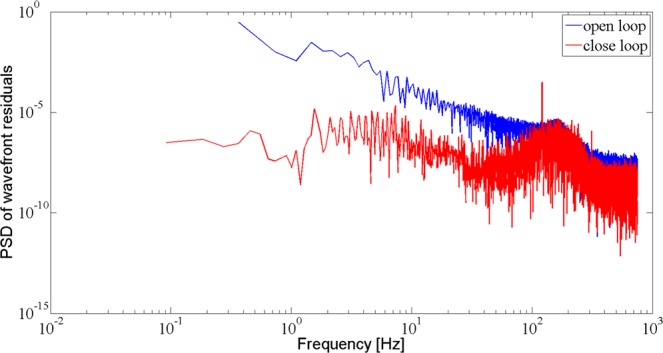
The PSD of the open and closed loop wavefront residuals.

Figure 7 shows the power spectrum density (PSD) of the open and closed loop wavefront residuals. The PSD data is recorded from the wavefront residual RMS value of the running AO system before and after correction with a recording telemetry of 1500 Hz. Figure 7 indicates that the CLCB of our AO system can reach about 120 Hz with an integral gain of 0.4 and a process delay of about 220 *μs*.

At the receiving part, the beams are reflected off to a CCD camera which allows monitoring the beam quality and AO system performance. The CCD is a 1024 × 1024 pixel HNu1024 with a 13 *μm* pixel size and operate with a 5 ms integration time. The focal length and the effective aperture of the zoom lens L3 are 814 mm and 80 mm respectively.

**Figure 8:**
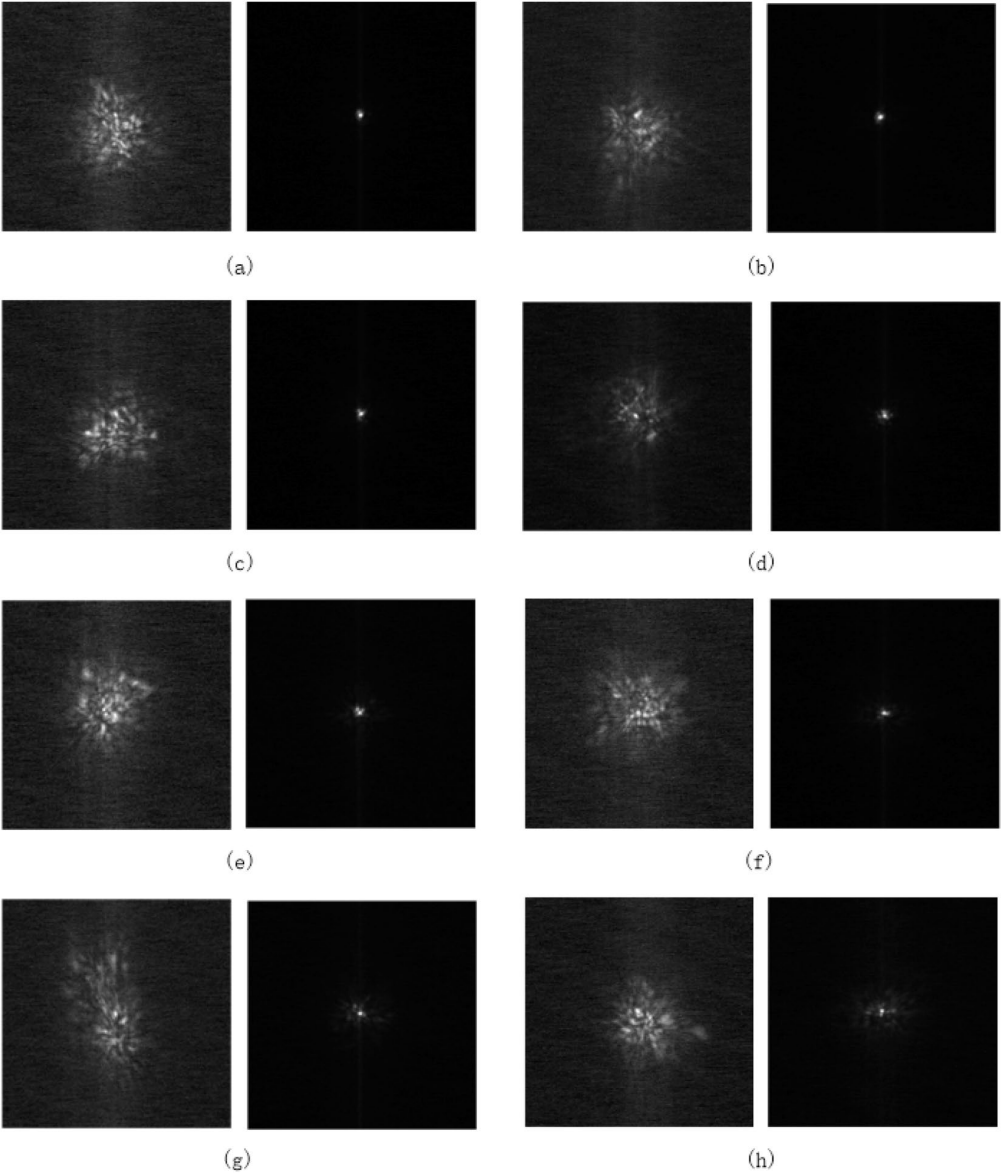
Far field image before and after compensation (*r*_0_ = 7 *cm*) with different Greenwood frequency, where are the images when *f*_*G*_ = 17.5 *Hz* to *f*_*G*_ = 144 *Hz* every 17.5 Hz from (**a**–**h**).

When the experimental system described above was setup, we can measure the mixing efficiency and BER value of the coherent FSOC system under different turbulence conditions. In this paper, we only verified the performance improvement under a fixed atmospheric coherent length and more turbulence conditions will be investigated in the future. In our experiment, the CLCB of this system was fixed at 120 Hz, and the simulative atmospheric coherent length *r*_0_ was 7 cm. Figure 8 shows the intensity image of the far filed for the experimental light beam under different Greenwood frequency. As shown in Fig. 8, when the AO system was off, the intensity distributions of the far field images were severely dispersive. On the contrary, after correction of the AO system, the intensity distributions of the far field were relatively centralized, and when the Greenwood frequency was low, it approached to the diffraction-limit. It was indicated that the AO system can compensate the wavefront distortion induced by atmospheric turbulence effectively. Besides, with the increase of Greenwood frequency, the temporal characteristics of the AO system had a serious impact on the laser energy concentration degree. Since the CLCB of the AO system was fixed at 120 Hz, a higher Greenwood frequency would lead to a worse performance of the coherent FSOC system.

**Figure 9:**
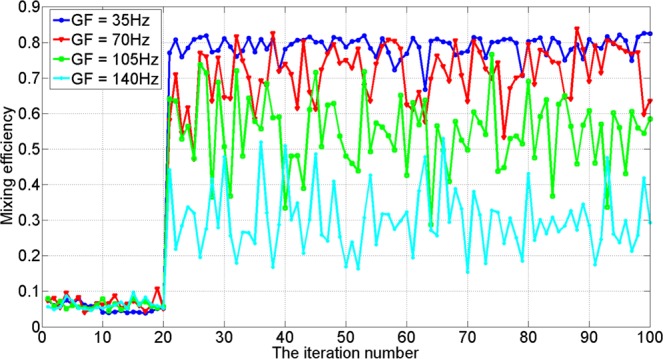
Mixing efficiency versus iteration number when *r*_0_ = 7 *cm* under different Greenwood frequency.

Under four different Greenwood frequency values, the mixing efficiency before and after AO correction are shown in Fig. 9. It can be seen that the mixing efficiency was below 0.1 under different Greenwood frequency without AO correction. After correction, the mixing efficiency was improved by different degrees under different Greenwood frequency. When Greenwood frequency equaled to 35 Hz and the AO system was on, the mean value of mixing efficiency was about 0.8, increased about 11 times compared with the mixing efficiency when the AO system was off. It was obvious that AO system can improve the mixing efficiency effectively. Meanwhile, with increase of Greenwood frequency, the mixing efficiency decreased dramatically.

**Figure 10:**
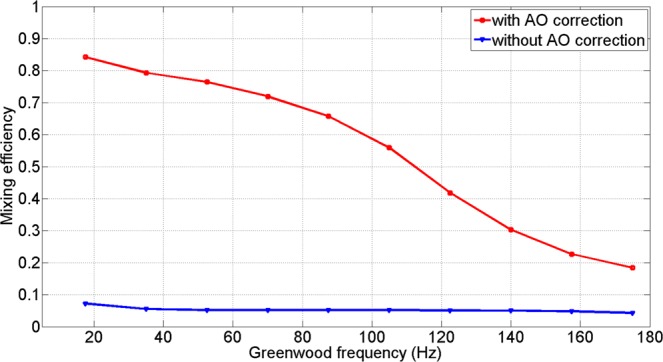
The experimental results of mixing efficiency under different Greenwood frequency when *r*_0_ = 7 *cm*.

Figure 10 illustrates the mean value of mixing efficiency of the experimental results under different Greenwood frequency. When Greenwood frequency was below 110 Hz, the average mixing efficiency can be larger than 0.5. And when Greenwood frequency was higher than 140 Hz, the mean mixing efficiency cannot reach above 0.3. That meant the dynamic ability of AO system cannot meet the need for Greenwood frequency higher than 140 Hz.

Compared with the simulated values as indicated in Fig. 1, the experimental values were lower than the simulated values. The causes of this difference include non-common optical path aberrations, the transmittance limitation of the lens and the DM fitting errors. Considering these error factors, the experimental results are consistent with the simulate results and the difference is acceptable.

**Figure 11:**
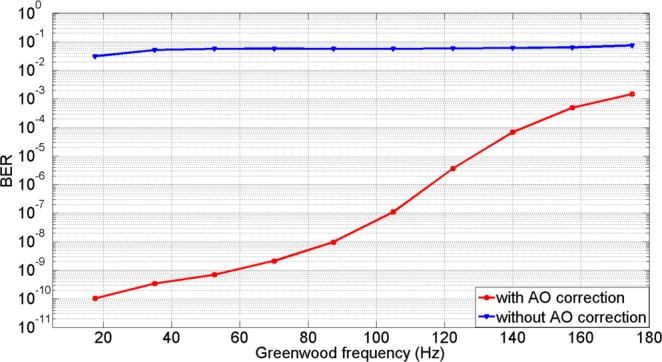
The experimental results of BER under different Greenwood frequency when *r*_0_ = 7 *cm*.

We assume the quantum efficiency *δ* is equal to 1 and the number of the photons per bit of received optical signal *N*_*p*_ is 12, according to Eq. 14 in Method section, Fig. 11 illustrates the system BER before and after AO correction. From Fig. 11, it was noted that the BER performance was severely degraded without AO correction. However, the values of BER were substantially improved after the AO system compensates for the wavefront phase distortion. When the Greenwood frequency was below 110 Hz, the values of BER decreased from around 10^−2^ to below or nearly 10^−6^. Due to the limitation of the CLCB, the values of BER could not be suppressed below 10^−6^ when the Greenwood frequency was larger than 120 Hz.

In order to give a quantitively performance improvement for 349-element AO system, we compared the performance of 349-element AO system with the 97-element AO system whose detailed results can be seen in ref.^13^. Since the maximum Greenwood frequency in ref.^13^ is 100 Hz. So, we intercepted the part of our experimental results that Greenwood frequency is less than 100 Hz for comparison.

**Figure 12:**
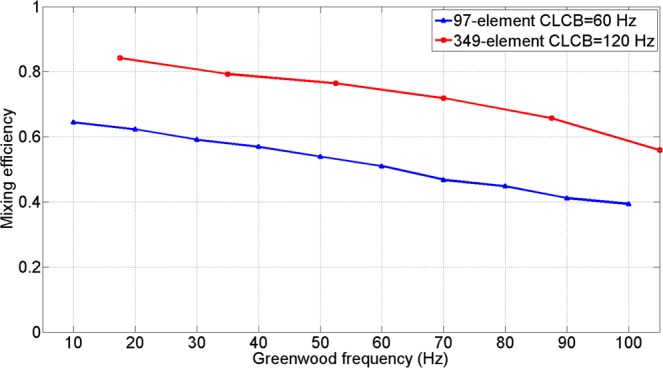
Comparison of mixing efficiency under different Greenwood frequency.

In the same condition of ${r}_{0}=7\,cm$, the comparative results with the 97-element AO system are shown in Fig. 12. In addition to the increase of element number, the CLCB of AO system was also improved to 120 Hz while the CLCB of 97-element AO system was 60 Hz. As shown in Fig. 12, the mixing efficiency had an increase of 0.2 on average under different Greenwood frequency. It was revealed that increasing the element number of DM and CLCB can offer more enhancements in coherent FSOC performance. And with the increase of Greenwood frequency, the temporal characteristics became the dominant factor that caused the performance degradation. Besides, since the *r*_0_ we simulate was 7 cm and the Greenwood frequency we simulated before was smaller than 100 Hz, so the high spatial and temporal frequency components of the simulative wavefront aberrations were still relatively small. Both of the two different AO system can get an acceptable result. But when the *r*_0_ gets smaller, the more element number are necessary to guarantee communication quality.

**Figure 13:**
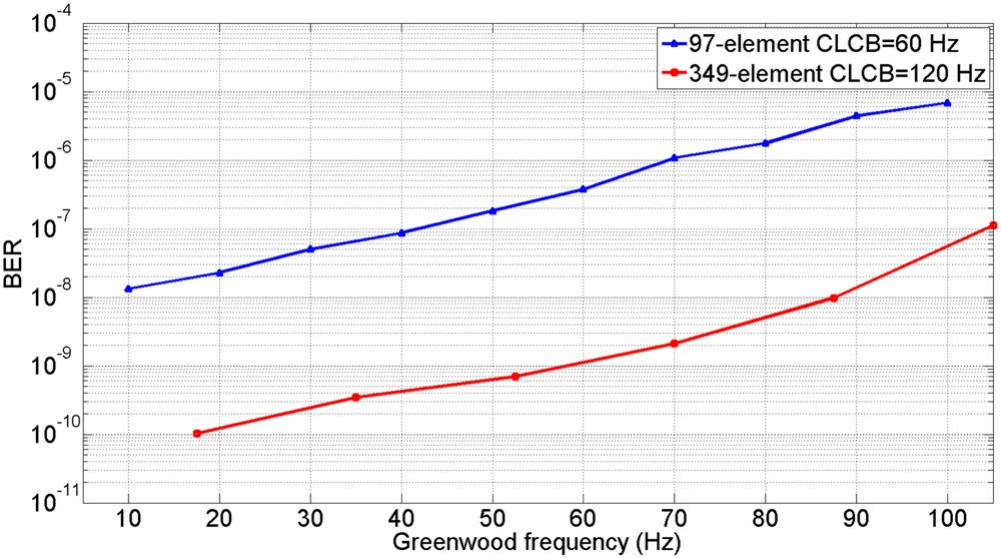
Comparison of BER under different Greenwood frequency.

Also, we can compare the BER performances of 349-element AO system with the 97-element AO system based on the same assumptions described above, as shown in Fig. 13. As one can see from Fig. 13, the values of BER decreased around two orders of magnitude on average. When the Greenwood frequency was 70 Hz, the BER value of 349-element AO system was nearly 10^−9^, while the BER value of 97-element AO system was only about 10^−6^. When the Greenwood frequency was below 20 Hz, the BER could even reach 10^−10^ after correction of 349-element AO system.

From the analysis of these experimental results, it is clearly that high-order AO system can significantly improve the performance of coherent FSOC system and more element number and higher CLCB can get more performance enhancement. For the same *r*_0_, the BER value of 349-element higher speed AO system decreased above two orders of magnitude compared with the 97-element AO system on average. And with the increase of Greenwood frequency, the CLCB of AO system became the bottleneck for the performance improvement of coherent FSOC system.

## Discussion

In this paper, we described a large-scale high-speed AO system with a 349-element deformable mirror to analyze the performance improvement for coherent FSOC system. For the first time, the performance improvement of coherent FSOC system using such a large scale AO system is quantitively verified by experimental results. Under different Greenwood frequency, the system mixing efficiency and BER improvement are analyzed which can offer an experimental reference or prediction for similar configurations of AO systems using for a given coherent FSOC system. The obtained experimental results showed that the value of BER can decrease two or three orders of magnitude when the element number of deformable mirror increased from 97 to 349 and the system servo bandwidth increased from 60 Hz to 120 Hz. In order to achieve favorable results (*BER* ≤ 10^−8^) in relatively severe turbulence conditions such as when the atmospheric coherent length was 7 cm and the Greenwood frequency was 80 Hz, the high order 349-element AO system should be employed since the low order AO system cannot guarantee the performance. In conclusion, our experiments have quantitively verified the performance improvement when a large-scale AO system is employed for coherent FSOC system. These experimental results can provide experimental reference in the parameters design of AO system used for coherent FSOC system.

## Methods

### The mixing efficiency

In the coherent FSOC system, the coherent detection involves an incoming signal combining with the light from a local oscillator (LO) beam coherently. Based on the theory of coherent detection, the total optical power of the combined beam is given by the expression^8^:

1 $$I={\int }_{S}\,\{{A}_{O}^{2}+{A}_{S}^{2}+2{A}_{O}{A}_{S}\,{\cos }\,[2\pi ({f}_{S}-{f}_{LO})+\Delta \phi ]\}ds$$

where Δ$\phi ={\phi }_{S}-{\phi }_{LO}$ is the phase difference between the optical signal and LO. *A*_*S*_ and *A*_*LO*_ represent the optical amplitudes of signal light and LO beam, and *f*_*S*_ and *f*_*LO*_ are their frequencies, respectively. In Eq. 1, when ${f}_{S}={f}_{LO}$, it is called homodyne detection, otherwise it is called heterodyne detection. Generally, a single symbol transmission time is less than 1 ns in a coherent FSOC system while the Greenwood frequency is in the millisecond scale, according to Taylor’s turbulence hypothesis, the phase aberrations caused by atmospheric turbulence can be considered frozen during the detection time in every code, then Δ$\phi$ is given by the expression:

2 $$\Delta \phi =\phi (r)+\phi (t)$$

where $\phi (r)$ represents the spatial part of phase aberrations caused by atmospheric turbulence which is time-indep-endent and $\phi (t)$ denotes the temporal part of phase in the optical signal, which is space coordinate-independent.

The mixing efficiency of homodyne detection is an important performance metric and defined by^5,8^

3 $$\eta =\frac{{[{\int }_{S}{A}_{S}{A}_{O}{\cos }(\Delta \phi )ds]}^{2}}{{\int }_{S}\,{A}_{S}^{2}ds\,{\int }_{S}\,{A}_{O}^{2}ds}$$

Because measuring the mixing efficency according to Eq. 3 is not easy, we can choose the Strehl ratio (SR) as a convenient approximate method to simplify the calculation, the detailed description can be seen in refs^8,20^.

Due to the temporal and spatial limitations, AO systems can partially compensate the turbulence-induced phase aberrations and some residual wavefront error is always left. According to Marechal approximation, the SR after AO correction can be estimated by the total residual mean square wavefront error value ${\sigma }_{\phi }$, which is given as^11,27^:

4 $$\eta \propto SR\propto {e}^{-{\sigma }_{\phi }^{2}}$$

There are three main sources of errors inherent in a closed loop AO system contributing to the total wavefront error and errors from each of the three sources can be treated as uncorrelated. Then the mean squared wavefront error is given by the following sum of individual error terms^11^:

5 $${\sigma }_{\phi }^{2}={\sigma }_{WFS}^{2}+{\sigma }_{fit}^{2}+{\sigma }_{time}^{2}$$

In Eq. 4, ${\sigma }_{WFS}^{2}$ is the wavefront measurement error arising due to the readout noise of the Shack-Hartmann wavefront sensor. ${\sigma }_{fit}^{2}$ is the spatial characteristics which represents the wavefront fitting error caused by the limited number of the deformable mirror actuators, it can be expressed by:

6 $${\sigma }_{fit}^{2}={\alpha }_{F}{(\frac{d}{{r}_{0}})}^{5/3}$$

where *α*_*F*_ is the fitting error coefficient which equals to 0.28 for CSDM^11^, *r*_0_ denotes the atmospheric coherent length, *d* is the equivalent interval of the actuators interval projected on the entrance pupil of the receiving antenna.

And ${\sigma }_{time}^{2}$ is the temporal error arising because of the contradiction between fast changing atmospheric turbulence and the finite CLCB of the AO system, it is given by^11^:

7 $${\sigma }_{time}^{2}=\kappa {(\frac{{f}_{G}}{{f}_{3dB}})}^{5/3}$$

where $\kappa$ is a constant which equals to 1 for the plane wave, *f*_3*dB*_ denotes the closed loop control bandwidth and *f*_*G*_ represents the temporal characteristic of turbulence, which is described as atmospheric Greenwood frequency and given by^11^:

8 $${f}_{G}={[0.102{(\frac{2\pi }{\lambda })}^{2}{\int }_{0}^{L}{C}_{n}^{2}(z){V}^{5/3}(z)dz]}^{3/5}$$

where *λ* represents the communication wavelength, *V* is the wind speed, ${C}_{n}^{2}$ denotes the refractive-index structure parameter and the integral range is the whole communication path.

Since EMCCD is widely used in wavefront sensors, and the readout noise of EMCCD is often lower than 1*e*^−^ with EM gain. So we only consider the spatial and temporal errors, then after AO compensation, the mathematical expectation of the residual wavefront phase aberrations based on a CSDM is given by^21,28^:

9 $$E({\sigma }_{\phi }^{2})=[{\alpha }_{F}{(\frac{d}{{r}_{0}})}^{5/3}+\kappa {(\frac{{f}_{G}}{{f}_{3dB}})}^{5/3}](rad)$$

Thus, the relationship between the mixing efficiency and the mean squared wavefront error can be expressed as^20,29^:

10 $$\eta \propto \exp \{\,-\,[{\alpha }_{F}{(\frac{d}{{r}_{0}})}^{5/3}+\kappa {(\frac{{f}_{G}}{{f}_{3dB}})}^{5/3}]\}$$

### The BER of coherent detection

In a coherent detection system, the BER value can be expressed as^13^:

11 $$BER=\frac{1}{2}erfc(\frac{Q}{\sqrt{2}})$$

where $Q=\sqrt{SNR}$ denotes the square root of signal-to-noise ratio (SNR) in coherent detection and the function *erfc* represents the complementary error function. For the synchronous binary phase shift keying (BPSK) coherent received system, the power of the signal light entering in the receive terminal is related to the amount of photons received within a single bit *N*_*p*_ as:

12 $${P}_{S}=\int \,{A}_{S}^{2}dS={N}_{p}h\nu B$$

where *h* is Planck constant, $\nu$ denotes the frequency of the communication laser and *B* represents the communication bit rate.

And the SNR without atmospheric turbulence is

13 $$SN{R}_{0}=\frac{2\delta {P}_{S}}{h\nu B}=2\delta {N}_{p}$$

where *δ* represents the quantum efficiency of the receiver detector. The signal power under atmospheric turbulence can be expressed as ${P^{\prime} }_{S}=\eta {P}_{S}$ and then the SNR can be expressed as $SNR=\eta SN{R}_{0}$ thus the BER of BPSK coherent detection is given by:

14 $$BER=\frac{1}{2}erfc(\sqrt{2\eta {N}_{p}\delta })$$

Thus, the relationship between the metric of coherent FSOC system performance and the parameters of AO system is described and the method description is complete.
